# Static and Impact Properties of Flax-Reinforced Polymers Prepared with Conventional Epoxy and Sustainable Resins

**DOI:** 10.3390/polym16020190

**Published:** 2024-01-08

**Authors:** Raffaele Ciardiello, Alessandro Benelli, Davide Salvatore Paolino

**Affiliations:** 1 Department of Mechanical and Aerospace Engineering, Politecnico di Torino, 10129 Turin, Italy; davide.paolino@polito.it; 2 Inter-Departmental Multi-Disciplinary Research Centre J-TECH @PoliTO, 10129 Turin, Italy; alessandro.benelli@polito.it; 3 Department of Applied Science and Technology, Politecnico di Torino, 10129 Turin, Italy

**Keywords:** bio-based resin, recyclable resin, composite materials, moisture, mechanical properties, impacts, sustainability, flax fibers

## Abstract

The study assessed the tensile, flexural, and impact properties of composite materials reinforced with flax fibers, employing three distinct resin types. The composite laminates were fabricated using three commercial resins: a conventional epoxy resin, an epoxy resin with a 31% weight concentration of bio-renewable content, and a recyclable methyl methacrylate infusion resin. This aims to assess if there exists a commercially available alternative to the traditional epoxy resin that can reduce the overall carbon footprint of composite materials. To investigate the influence of humidity on the mechanical behavior of the flax layers, a drying treatment was applied to the fibers before the infusion process. Micro-computed tomography analysis revealed that heat treatment resulted in a reduction of porosity, although it did not affect the mechanical response of the composite laminates. Moreover, laminates produced with non-recyclable and sustainable resins exhibited no significant change in tensile and flexural modulus. In contrast, those produced with recyclable resin demonstrated a slight reduction in the strengths of the composite laminates. Conversely, out-of-plane impact tests and repeated impact tests indicated that composites prepared with recyclable and bio-epoxy resin formulations present superior damage resistance to repeated impact compared to traditional epoxy resin.

## 1. Introduction

In recent years, there has been a significant increase in interest towards sustainability in industry, driven by growing concerns about pollution and climate change [1]. Consequently, industries such as automotive and aerospace, which face stricter emissions regulations [2,3], are increasingly adopting lightweight materials and promoting sustainable design principles. Composite materials are considered ideal lightweight materials due to their high specific strength compared to metallic materials. As a result, composites are gradually replacing metals in various applications, significantly reducing weight without compromising structural integrity and the possibility of obtaining multifunctional materials [4,5,6].

Among composite materials, thermoset composites are widely used for structural applications. They are designed in different configurations (e.g., unidirectional fibers or fabrics) and with various reinforcements like glass fiber or carbon fiber, to meet specific mechanical requirements [6]. While thermoset composites contribute to weight reduction and emission reduction, concerns about their full sustainability persist. This is because thermoset resins and fibers are not fully recyclable and are primarily derived from petroleum [7]. The manufacturing process of thermoset composites is thus not entirely sustainable and eco-friendly. For instance, the synthesis of epoxy resin, a common thermoset matrix, has a significant environmental impact and contributes to the carbon footprint and greenhouse emissions in the automotive industry [7]. For this reason, regulations have been imposed to limit the use of petroleum-based and non-renewable components [8,9].

One potential solution to address these concerns is the substitution of thermoset matrices with recyclable or renewable alternatives. However, thermoplastic composites are not a viable solution as they possess lower mechanical properties, which do not meet the required strength and safety standards for most structural applications [10]. Additionally, the glass transition and melting temperatures of thermoplastic polymers can limit their industrial use [11,12,13]. In recent years, the polymer industry and research community have focused on developing specific formulations to achieve good mechanical performance, chemical stability, and thermal stability as composite matrices [11,12]. Several studies have highlighted that around 80% of polymer materials used in composites are derived from non-renewable fossil resources, underscoring the importance of replacing thermoset matrices with renewable and bio-based components to contribute to decarbonization goals [11,12].

Numerous studies in the literature have compared the mechanical properties of bio-based resins with those of epoxy resins to evaluate their performance. Derahman et al. [14] compared composite materials fabricated with a bio-based resin (derived from Jatropha seed) to synthetic epoxy resin. The mechanical tests revealed that the tensile and flexural properties of the bio-based resin composites were not comparable to epoxy resin composites, and the addition of bio content also had a detrimental effect on epoxy resin properties. Gour et al. [15] investigated the mechanical and chemical behavior of composites with varying percentages of two bio-based resins (cardanol-based) mixed with epoxy resin. The results showed that the addition of bio-based resin led to increased absorbed energy in Izod impact tests and a slight increase in ultimate load in tensile tests. However, the specific bio-based resin and its concentration influenced the mechanical properties differently. Terry and Taylor [16] demonstrated that fully bio-based monomers could adversely affect mechanical properties and glass transition temperature. Partially bio-based epoxies were found to be a viable alternative for vacuum-infused composites in the automotive, marine, aerospace, and wind energy industries. The study identified systems with certain bio-content percentages that exhibited comparable strength, stiffness, and toughness to traditional epoxy resins. Nikafshar et al. [17] explored the mechanical properties of vanillin-based epoxy resins, considering the environmental impact of traditional epoxy resin, bisphenol A diglycidyl ether (DGEBA). The results showed enhancements in tensile strength and impact strength for certain vanillin-modified epoxy compounds. However, a decrease in glass transition temperature could limit their application in certain industries. Ma et al. [10] utilized itaconic acid as an alternative feedstock to prepare bio-based epoxy resins, which demonstrated higher mechanical and thermal properties compared to DGEBA.

Pursuing an alternative perspective, different classes of thermoplastic matrixes for composite materials have been studied and developed in recent years due to their re-processability. One promising thermoplastic resin is Elium^®^ thermoplastic resin. Elium is a reactive methylmethacrylate-based fully recyclable resin. At ambient temperature, it is in the liquid state and presents low viscosity. Due to this peculiarity, it can be used to produce composites with a vacuum-assisted resin infusion technology. Thus, composite material components can be manufactured by using the same processes developed for thermoset infusion resins. Further, its enhanced recycling capabilities have been demonstrated by several studies [18,19,20].

Several studies have already been carried out on the mechanical performance of the Elium thermoplastic resin [21,22,23,24]. They showed that the Young modulus and the flexural and tensile strengths of the Elium composites are comparable with the epoxy composites, although the Elium composites withstand greater deformations at failure due to their viscoelastic response.

Several authors investigated the impact response of Elium thermoplastic reinforced with different fibers (glass, carbon, UHMWPE) [25,26,27]. All these authors confirmed that the composites made with ELIUM resin present enhanced impact resistance compared with composite materials prepared with epoxy resin. Further, they showed that the impact strength, fracture toughness, and energy absorption before the onset of the major failure of ELIUM composites are significantly higher when compared with conventional epoxy composites. Furthermore, this material has also been used to weld composite materials, improve damping properties, evaluate recycled properties, and improve fatigue capability [28,29,30,31,32,33,34,35]. One drawback is related to the manufacturing process of Elium resin that cannot be infused at high pressure since the process can trigger boiling of the resin and thus void formations [36]. However, Ciardiello et al. [37] designed a fabrication methodology that allows infusing composite laminates at 0.8 bar with low porosities < 1%.

While the mechanical properties of bio-based resins appear promising, further research is necessary to compare them with commonly used epoxy resins in composite materials, ensuring safe design and structural integrity. Moreover, the suitability of these resins for vacuum infusion processes should be investigated due to potential viscosity issues [13,38]. Mechanical characterization of bio-based composite laminates is of utmost interest to universities and industries as it represents a promising approach for reducing the carbon footprint while employing existing production technologies.

The use of flax fibers has been investigated in the last 10 years due to their good mechanical properties and lower environmental impact. However, these fibers present different problems compared to synthetic fibers (i.e., glass or carbon fibers) such as the moisture absorption of the fibers, which affects the mechanical properties [39,40,41,42,43,44] of the composites. Moreover, the mechanical, physical, and chemical properties are strongly dependent on the harvesting conditions such as climate, location, soil characteristics, and weather properties [45,46]. Finally, another observed issue in composites reinforced with natural flax fibers is related to the poor interface quality between the fibers and the polymer matrix. To improve the adhesion between these two components, chemical pre-treatments are frequently employed. Though a final optimal treatment has not yet been found, one of the cheapest solutions is the alkalization of the fibers [45], which has been regularly adopted.

In this work, three commercial resins have been used to infuse composite laminates made with flax fibers. Tensile, flexural, and impact tests were carried out to compare the mechanical behavior of composite materials made with a recyclable resin, a traditional epoxy resin, and a partially renewable epoxy resin. The flax fibers have been infused by considering two different conditions, dried and undried, to assess the effect of humidity on the laminate response [39,40,41,42,43,44]. The flax fibers do not present any pretreatment. Furthermore, the porosity concentration has also been quantified with micro-CT analyses and its effect on the mechanical properties of the composite laminates has been investigated in the paper. The work aims at assessing and comparing the mechanical properties of flax composite laminates prepared with three different commercial resins to find an alternative solution to conventional epoxy resin, which still presents a high environmental impact. Thus, the activity provides a comparison of available alternative solutions to reduce the carbon footprint of composite materials. Furthermore, the work illustrates that drying the fibers before infusion can be worthless due to the quick retention of moisture. For this reason, a complex fabrication methodology should be studied to avoid the presence of moisture during infusion. A viable solution to this issue can be the correlation among the relative humidity of the environment, the moisture wt.% absorbed by the fibers, and the mechanical properties in order to evaluate whether the obtained results can fit with the specific applications. Avoiding the drying treatment contributes also to lowering the carbon footprint.

## 2. Materials and Methods

### 2.1. Materials, Methods and Laminate Production

#### 2.1.1. Materials

Three different infusion resins were adopted in this work to laminate flax-reinforced laminates. Composite laminates were fabricated employing three distinct resins: a conventional epoxy resin designated as IN2 (EasyComposite Ltd., Stoke-on-Trent, UK), an epoxy resin with a 31% bio-based composition denoted as IB2 (EasyComposite Ltd., Stoke-on-Trent, UK), and a recyclable thermoplastic poly-methyl methacrylate, Elium by Arkema (Colombes, France). The bio-based nature of the IB2 resin derives from the utilization of plant-derived glycerol instead of petroleum-based propylene. Furthermore, the epichlorohydrin component in the bio-based epoxy resin is synthesized using renewable plant-based glycerol as a substitute for petroleum-based propylene. Both epoxy and partial bio-epoxy resins use an amine hardener with a resin/hardener ratio of 100:30 and 100:22, respectively. On the other hand, the methyl methacrylate resin uses a water-free benzoyl peroxide (BPO) hardener with a resin/hardener ratio of 100:3. The values of the densities, viscosities, and tensile and flexural properties are reported in Table 1. These values are drawn from the datasheet.

**Figure 1:**
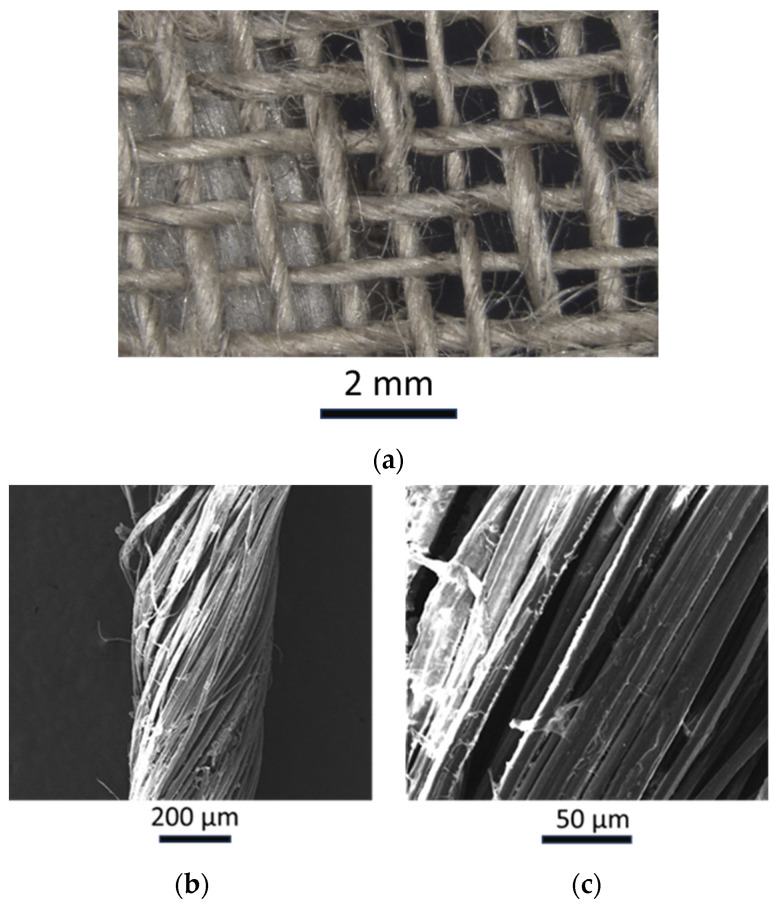
(**a**) optical image of the woven fabric; (**b**) SEM analysis of a yarn at a magnification of 200×; (**c**) SEM analysis of a yarn at a magnification of 200×.

Woven balanced (2 × 2) flax fibers (FLAXDRY BL200, Valliquerville, France) were used as reinforcement. The fiber fabrics present a fiber density of 1.27 g/cm^3^, a surface density of 220 g/m^2^, and a similar yarn structure along warp and weft (10.2 yarns/cm and 10.1 pick/cm, respectively). The technical datasheet [50] reports a tensile strength of 60 MPa and 6.5 GPa, in both warp and weft directions, respectively, of composite made with these fibers and an epoxy-based system but it does not report the mechanical properties of the fibers themselves. No pretreatment was carried out by the supplier. A microscopy analysis was carried out on the fiber to show the geometry of the 2 × 2 woven fabric. Figure 1a shows a microscopy analysis of the flax fabric. As reported by Charlet et al. [51] and Coreller et al. [52], the yarn section of the flax fiber is not uniform. The microscope analysis showed that the yarn section presents diameters from 270 to 400 µm. Figure 1b and Figure 1c report the SEM micrographs of a single yarn at 200× and 1000× magnification, respectively. Figure 1b shows a good distribution of the fibers in the yarn, whereas Figure 1c shows that the single fiber presents a diameter from 9 to 15 µm. The SEM analysis was carried out by using a Field-emission Scanning Electron Microscopy (FeSEM), Tescan Mira3 (Brno, Czech Republic). An accelerating voltage of 15 kV was used together with a secondary emission signal. The specimen surfaces were coated with gold. A Zeiss Microscope AX1O (Jena, Germany) was used for optical images.

#### 2.1.2. Fabrication of the Laminates

**Figure 2:**
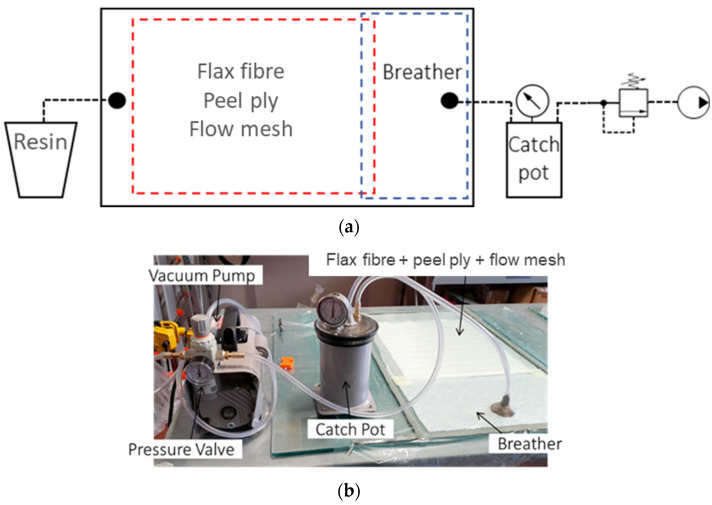
(**a**) Sketch of the infusion process; (**b**) Adopted infusion configuration.

Figure 2a,b illustrate the refined infusion configuration utilized in the VARTM (Vacuum Assisted Resin Transfer Molding) process conducted within the laboratory of Politecnico di Torino. In particular, Figure 2a shows a representative sketch of the infusion configuration and Figure 2b the actual infusion configuration. As demonstrated by Ciardiello et al. [37], this suggested setup is well-suited for producing composite laminates without observable imperfections by using Elium resin. The infusion process was conducted at room temperatures ranging from 21 to 23 °C.

A glass plate was used as the bottom mold. The glass surface was prepared with a releasing wax to facilitate easy separation. Four layers of 2 × 2 twill fabric flax fiber, each with a consolidated average thickness of 0.6 mm, were arranged over the glass mold with attention to aligning the weft fibers. A peel-ply and a flow mesh fabric were subsequently applied over the flax fiber. The role of the flow mesh was to ensure a consistent and uniform resin distribution during the infusion process, while the peel ply facilitated the quick separation of the final laminate from the upper layer.

A breather layer was positioned before the resin outlet, as depicted in Figure 2a. For a 350 × 350 mm laminate, the dimensions of the breather layer measured 200 × 350 mm. The breather cloth, composed of non-woven polyester fabric, was specifically designed to facilitate airflow throughout the vacuum bagging process. However, it should be noted that the breather mesh had a finer texture compared to the flow mesh, which induces the braking of the resin flow. In this instance, the breather layer served a dual purpose: it regulated resin flow by slowing it down upon contact, thereby ensuring an even resin distribution over the fiber layers.

Upon complete saturation of the flax fibers with resin, the resin outlet was sealed. After a 3-min interval, the resin outlet was once again sealed to allow for resin consolidation within the bag. A catch-pot was connected to the resin outlet via a silicone tube to prevent the resin from reaching the pump. Between the catch-pot and the vacuum pump, a pressure-regulating valve was installed to tune the infusion pressure. The resins, after mixing, were degassed for 4 min before the infusion. After the infusion, the resin curing took place at room temperature for 24 h. Finally, the laminates made with ELIUM and IB2 resins underwent a post-curing phase in an oven for one hour at 80 °C while the ones made with IN2 were cured for three hours at 100 °C, as suggested by the manufacturers. Six composite laminates were prepared by using the three resins and both dried and undried fabrics. Five specimens for tensile tests, six specimens for flexural tests, and three specimens for impact tests were used and considered for the statistical analysis.

#### 2.1.3. Dried and Undried Laminates

**Figure 3:**
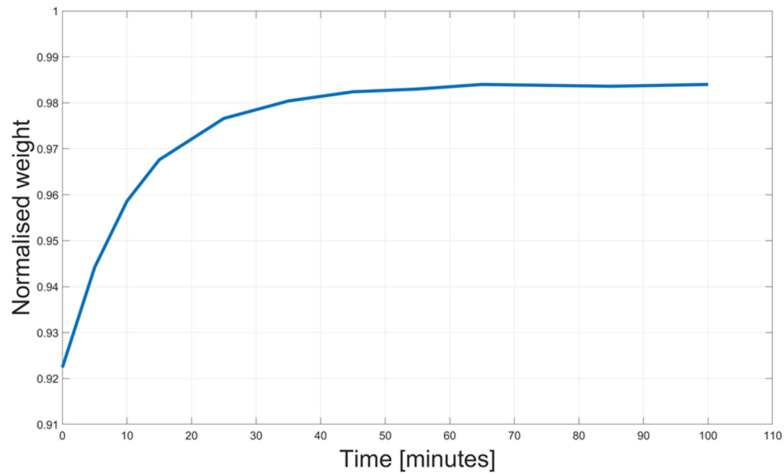
Retained moisture weight.

The composite laminates prepared with the dried and undried flax fibers were fabricated by using a similar procedure presented by Maudood et al. [44]. Both dried and undried flax fiber fabric pieces were oven-dried at 60 °C for 8 h to ensure identical initial moisture content. Then, four layers of fabric were placed on the mold, covered with peel ply and flow mesh and left in the lab environment for 8 h at the relative natural humidity between 30% and 40% to reach equilibrium with the RH level of the environment before the infusion. On the other hand, the dried fibers were sealed on two sides with the vacuum bag, and the remaining two sides were left open in the oven at 60 °C. After 2 h, the bag was completely sealed and put under vacuum. Then the mold was removed from the oven and the infusion was carried out when the room temperature was reached. Preliminary tests were carried out on five specimens to assess the moisture content before infusion quantitively. The results of this preliminary activity are shown in Figure 3. Squared flax fabrics, 40 × 40 mm, were weighed and then conditioned at 60 °C for 8 h. After 8 h, the squared samples were kept out of the oven and their weight was monitored for 100 min to assess the retained weight that can be directly associated with the moisture in the flax fibers. Figure 3 shows that the flax fabrics present an average moisture content of 6.1 wt.%. since, after the heating cycle, they present a loss of weight of 6.1%. Maudood et al. [40] found a similar content in their work. At an RH level of 40%, the moisture content they found was 5.5%. Then, once they are removed from the oven, they quickly regain the moisture. Figure 3 shows that up to 98% of the original weight is regained in 25 min. This is also the reason why the drying cycle and vacuum were performed in the oven.

### 2.2. Mechanical Properties

#### 2.2.1. Tensile Tests

The tensile properties of composite specimens fabricated with three different resins were investigated. The experiments were conducted using an Instron 8801 testing machine (Instron, Norwood, MA, USA) equipped with a 100 kN load cell. The tests were performed by imposing a crosshead displacement rate of 2 mm/min. Strain gauges were placed on the specimens to determine the strain in both the longitudinal and transversal directions, which allowed for the subsequent computation of Young’s modulus. Rectangular cross-section specimens, with a nominal width of 25 mm and a length of 250 mm, were tested following the guidelines provided by ASTM D3039 [53]. The actual width and thickness of each specimen were measured with a digital caliper possessing a resolution of 0.01 mm in order to accurately compute the applied stress during the tests.

#### 2.2.2. Flexural Tests

Flexural tests were conducted following the guidelines of ASTM standard D790 [54]. The test specimens present a rectangular cross-section with a nominal width of 12.7 mm. A support span of 38.4 mm, calculated as sixteen times the average thickness of the specimens, was used. The test speed was set at 1.0 mm/min, taking into account the average specimen thickness and the support span. Flexural strain and stress were calculated according to the standard and the beam theory reported in Equations (1) and (2).

(1) $$\left(\varepsilon =\frac{Dd}{L^{2}}\right)$$

(2) $$\left(\sigma =\frac{3PL}{{\left(2bd\right)}^{2}}\right)$$

In the strain and stress equations, *P* represents the measured load, *L* denotes the support span, *b* is the specimen’s width, *d* indicates the specimen’s thickness, and *D* corresponds to the maximum deflection at the beam’s center.

#### 2.2.3. Impacts

Impact testing was conducted to assess the out-of-plane impact response of the composite laminates. These tests were carried out according to the ASTM D5628 standard [55] by using the FractoVIS (Instron, Norwood, MA, USA) free-fall drop dart testing machine.

Squared specimens, measuring 100 × 100 mm, were used for this activity. The specimens displayed varying thicknesses, ranging from 2.30 to 2.42 mm. Notably, the clamping system of the drop tower uses a 76 mm ring designed to apply pressure to the laminate to avoid the slippage of the specimen during the impact.

The impact energy can be set by using both the falling height and the impacting mass. The impacting dart presents a cylindrical shape with a hemispherical tip of 20 mm in diameter. A total falling mass of 5.89 kg was adopted, and an impact energy of 25 J was used for carrying out perforation tests. This energy level was selected following preliminary tests to achieve complete specimen perforation. The impact force is measured through a piezoelectric load cell, positioned at the upper end of the dart, and load data were acquired at a frequency of 1 MHz. During the falling of the dart, some potential energy may dissipate due to friction. Therefore, the energy balance equation (as depicted in Equation (3)) must account for this non-conservative term, denoted as *W_f_*.

(3) $$E_{0}=mgh=\frac{1}{2}mv_{p}^{2}+W_{f}\to W_{f}=mgh-\frac{1}{2}mv_{p}^{2}$$

The variables of the Equation (1) are reported below:

“m” represents the mass of the falling object,

“g” is the gravitational constant,

“h” is the initial height from which the object falls,

“$W_{f}$” accounts for the non-conservative work attributed to friction,

“$v_{p}$” corresponds to the velocity measured by an electro-optical device, specifically a photocell, capturing the dart’s velocity at the precise moment of impact.

It is noteworthy that this device also serves as the trigger for initiating the load data acquisition process. Additionally, the displacement of the dart during the impact event is determined by performing a double integration of the acceleration data, obtained through the application of Newton’s law, reported in Equation (4). Then, Equation (4) is integrated as reported in Equation (5), where the velocity at impact is added to account for the velocity at the begin of the impact event. Finally, v(t) is integrated as reported in Equation (6).

(4) $$a\left(t\right)=g-\frac{F\left(t\right)}{m}$$

(5) $$v\left(t\right)=\int_{t_{0}}^{t_{f}}a\left(t\right)dt+v_{p}$$

(6) $$s\left(t\right)=\int_{t_{0}}^{t_{f}}v\left(t\right)dt$$

In Equations (4)–(6), F(t) is the time history of the force signal acquired by the load cell at the time t, $t_{f}$, and $t_{0}$, that are, respectively, the last and the first-time instant of the impact; v(t) and s(t) are, respectively, the velocity and the displacement of the dart calculated at the time t.

The energy $E_{ab}$ absorbed by the specimen during the impact corresponds to the area under the force-displacement curve:

(7) $$E_{ab}=\int_{s_{0}}^{s_{f}}F\left(s\right)ds$$

where $s_{f}$ and $s_{0}$ are the dart positions evaluated, respectively, at $t_{f}$ and $t_{0}$.

Perforation tests and impact tests were carried out in this analysis. A Damage Index, proposed in the literature by Belingardi et al. [56], was used to monitor the damage progression of composite laminates subjected to repeated impacts. The damage index is defined as:

(8) $$DI=\frac{E_{a}}{E_{i}}\;\frac{s_{max}}{s_{i}}$$

where $E_{a}$ and $E_{i}$ are the absorbed energy during the repeated impacts and $E_{i}$ is the absorbed energy with the perforation test. $s_{max}$ and $s_{i}$ are, respectively, the maximum displacement of the laminate during the repeated impacts and the maximum displacement in the perforation impact test. *DI* assumes values between 0 and 1 when the specimen is undamaged and fully damaged. Thus, this index is used to monitor the crack propagation during repeated impact test campaigns. The last impact usually presents a value close to 0 or greater than 1 due to the lack of mechanical resistance before failure or to the friction between the dart and the hole generated in the composite materials. For this reason, the *DI* was set to 1 in these cases, when complete failure was observable in the specimen.

### 2.3. Tomography

X-ray computed tomography (CT) was performed using a custom-made CT system located in Politecnico di Torino J-Tech@PoliTO laboratory (Torino, Italy). The facility is equipped with a 300 kV X-ray source and a 5 μm minimum focal spot size, along with a flat panel detector featuring 2048 × 2048 pixels. The working distance between the source and the sample, as well as between the source and the detector, can be adjusted as needed. The CT system has been used at high magnifications since de Kergariou [41] showed that both voids in the resin and within the fibers can be detected. Six specimens were analysed for each laminate configuration. The scanning parameters used were 80 kV and 110 µA, resulting in an electron beam power of 8.8 W and a final resolution of 13 μm per voxel. No physical filtering was applied to the X-rays. The reconstructed 3-D volume of the investigated specimens was obtained using the filtered back-projection algorithm through VG MAX 3.5 software (Volume Graphics GmbH, Heidelberg, Germany), utilizing a total of 1600 X-ray projections. During post-processing, the fibers were successfully separated from the matrix material, and the defects present in the different laminates were detected. Finally, the porosity of the laminates was computed.

## 3. Results and Discussion

### 3.1. Tomography

#### Porosity Analysis

**Figure 4:**
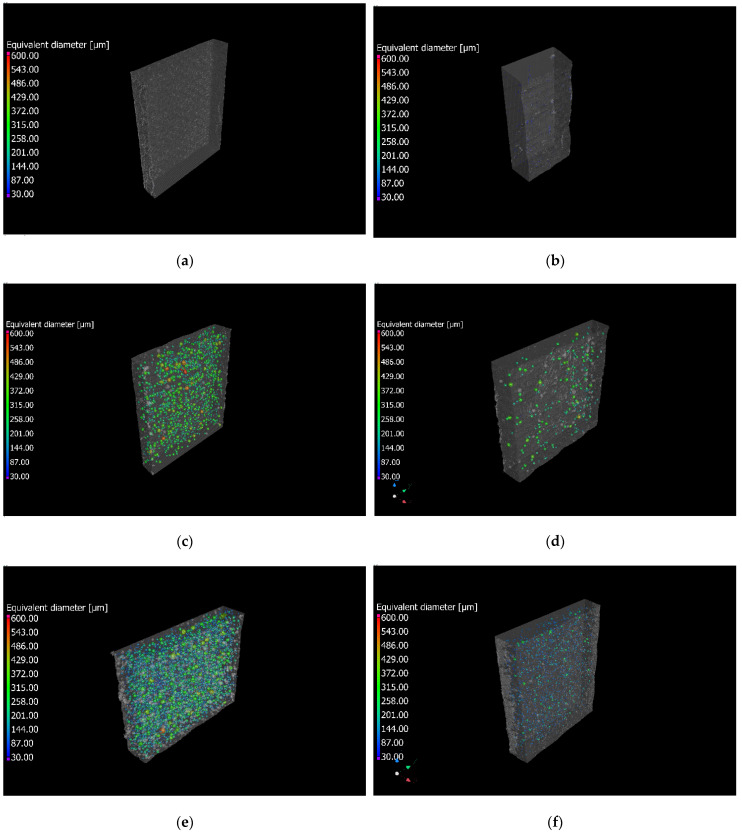
Representative void distributions of the composite laminates produced with different resin systems and fiber treatment. (**a**) Elium fabricated with undried fibers; (**b**) Elium produced with dried fibers; (**c**) IN2 fabricated with undried fibers; (**d**) IN2 fabricated with dried fibers; (**e**) IB2 produced with undried fibers (**f**) IB2 fabricated with dried fibers.

Figure 4a–f depict the representative populations of voids in each specimen. In particular, Figure 4a,c,e report the CT images of the composite laminates fabricated with undried fibers. Figure 4b,d,f show the CT images of the composite laminates fabricated with the dried fibers. The total porosities for each laminate are presented in Table 2. The colour bars were chosen to highlight the majority of the porosities.

A decrease in the porosity can be observed for the composite materials prepared with dried fibers both for IN2 and IB2. On the other hand, the porosity of Elium resin is almost equal to zero in the observed specimens for the dried and undried laminates. Table 2 reports a 0% porosity for composite laminates made with Elium resin due to the resolution of the µ-CT analysis, which does not permit detection of pores with diameters below 12 µm. The porosity reduction after the drying cycle was approximately 80% for the IN2 resin and around 90% for the IB2 resin. This reduction can be attributed to the absorption of flax fibers of water from the relative humidity of the environment. Although some works relate the presence of porosity to the presence of moisture [40,42] in the fibers, the mechanism is not well defined and studied in the literature to the authors’ best knowledge. However, different works [40,42] showed that the presence of voids increases with the moisture content in the flax fibers. It seems possible that the voids are generated from the presence of moisture in the fibers when the exothermic curing starts. Thus, the exothermic reaction raises the temperature of the fibers and triggers the evaporation of the moisture, thereby generating the voids in the matrix if the viscosity and the density are high enough to lock the voids and avoid floating of voids in the resin. The CT analysis was also used to assess the resin contents. All the resins present a similar resin and fiber content, which ensures an appropriate comparison among the mechanical properties of the different composites. The fiber content is 30–31% for all the composite laminates.

### 3.2. Tensile Tests

**Figure 5:**
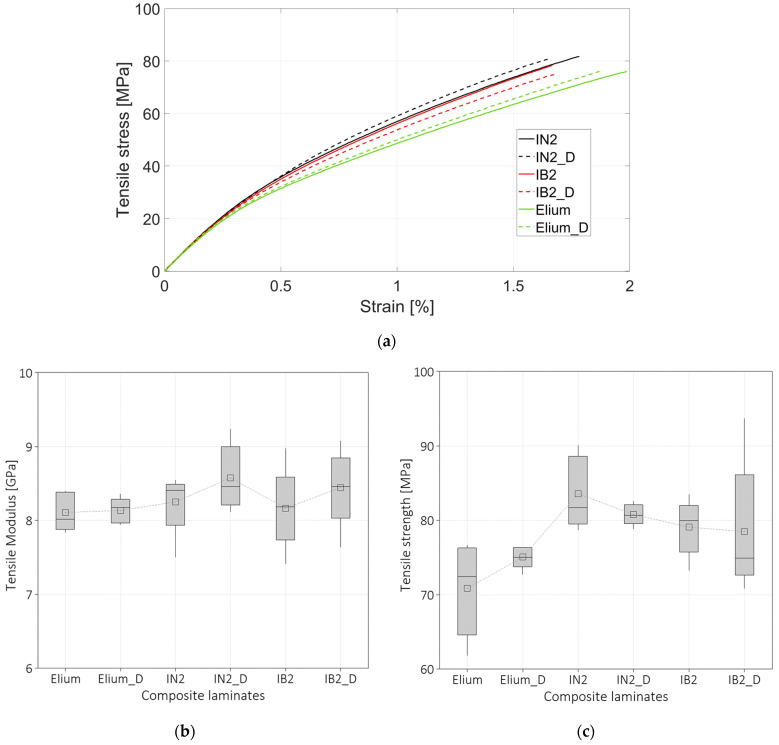
(**a**) Representative stress-strain curves of the tensile tests; (**b**) Box plot of the tensile modulus; (**c**) box plot of the tensile strength.

Figure 5a compares the representative stress-strain curves obtained from experimental tensile tests for all six composite laminates prepared with the resins IN2, IB2, and Elium. A letter D has been added to the nomenclature for the laminates prepared with the dried fibers. The strain was measured by using linear strain gauges (HBM 1-LY48-3/350) purchased from HBM (Darmstadt, Germany) and installed in the middle of the tested specimens and acquired with an acquisition board (NI 9237) from National Instruments (Austin, TX, USA). Figure 5 shows that specimens IN2 and IN2_D present slightly higher ultimate stress compared to all the laminates prepared. All the curves related to the composites prepared with different resins and drying treatments present the same initial trend up to 15 MPa and thus similar Young’s moduli. The Young’s moduli were computed with a trendline in the interval 0–15 MPa since the curves deviate from linearity after 15 MPa. This behavior is more evident for the Elium curves and it is likely due to the lower Young’s modulus of this resin, which is 2.6 GPa as reported in Section 2.1.1. Further, all the composite materials prepared with different resins present curves that can be represented by using a bi-linear trend. The first initial trend is representative of Young’s modulus, and the second one is maintained up to failure. This is a typical behavior of composite laminates made with flax fibers [57]. Concerning the strengths, the composite laminates prepared with IN2 resin present higher strengths compared to Elium and IB2, which present similar values.

The summary of the results related to Young’s moduli and maximum strengths are reported in Figure 5b and Figure 5c, respectively. Figure 5b illustrates that there are no significant differences in Young’s moduli among the composite laminates prepared with the different resins and pretreatment. Analysis of Variance (ANOVA) was carried out throughout Minitab 21.4 (State College, PA, USA) software to determine whether there are statistical and significant differences among the properties obtained with different composite materials. The one-way ANOVA analysis confirmed that there is no significant difference in the Young’s modulus. A $P_{value}=0.428$ was found and a significance level α=0.05 was considered. In ANOVA analysis, the null hypothesis that all the means are equal is rejected if the *P_value_* is lower than α.

Figure 5c reports the values of the strengths for all the analysed composite laminates. The box plot shows that the strength of the composite made with Elium resin is lower compared to the composite prepared with IN2 and IB2 resins. On the other end, the maximum and minimum values of the box plot related to IB2 and IN2 resins show that there is not a significant difference. However, ANOVA analysis showed that there is a significant difference in the strength likely due to the lower values of the composite fabricated with Elium resin. For this analysis, a $P_{value}=0.012$ was found and a significance level α=0.05. Indeed, if a mean value is computed between all the IN2 and IB2 composite-based laminates and compared with the mean values of Elium and Elium_D, Elium strength results 8% lower compared to IN2 and IB2 composite laminates. A significant difference in the final tensile strain was not observed for the composite prepared with dried and undried fibers.

### 3.3. Flexural Tests

**Figure 6:**
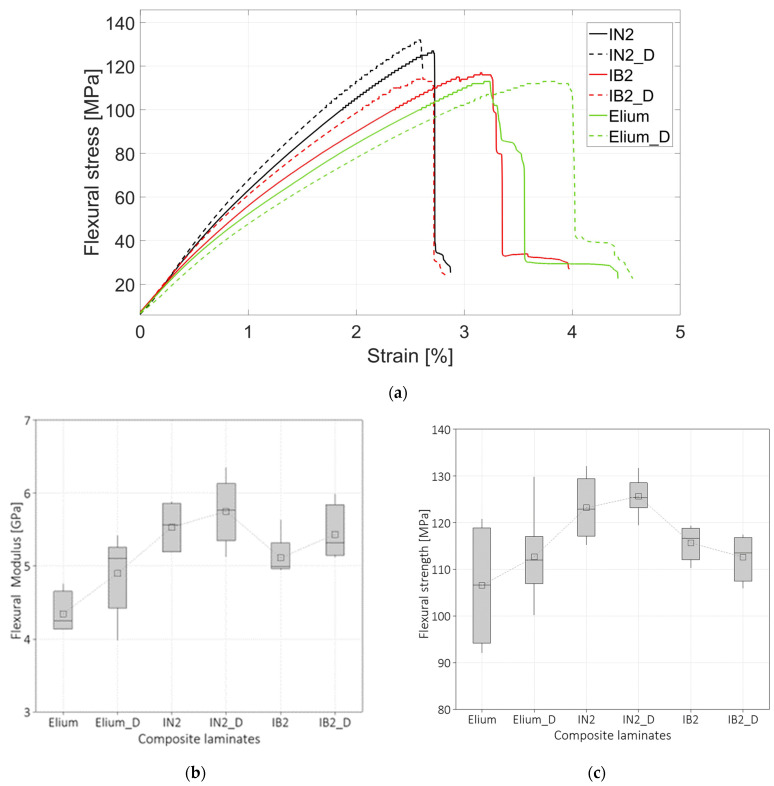
(**a**) Representative stress-strain curves of the flexural tests; (**b**) Box plot of the flexural modulus; (**c**) box plot of the flexural strength.

The representative stress-strain curves obtained from flexural tests for the six composite laminates prepared with the resins IN2, IB2, and Elium are compared in Figure 6a. The nomenclature for the laminates prepared with dried fibers includes the addition of the letter D. The curves depict a similar trend to the tensile curves. The trends look again bi-linear for IB2 and ELIUM resin while the composite prepared with IB2 resin is more linear compared to the other composite materials. The curves show also that the strains at failure for composites prepared with IB2 resin and ELIUM resin are larger compared to the composite prepared with the conventional epoxy resin. This behavior was also observed by Boursier et al. [38] and Iadarola et al. [13], who showed that the resins that have a higher bio content exhibit a larger deformation to failure both for flexural and tensile tests. Flexural tests conducted on composite laminates prepared with flax fibers also showed that the strain to failure increases for both composites prepared with IB2 and Elium resins. Furthermore, the composites prepared with Elium and IB2 resin present a more ductile behavior compared to the laminates prepared with the IN2 resin. One-way ANOVA shows that there is a significant difference for both flexural modulus and strength. $P_{value}=0.0$ and $P_{value}=0.005$ were found for flexural modulus and strength respectively. The box plots in Figure 6b and Figure 6c illustrate the differences in the flexural moduli and strength, respectively, for the six laminates. The flexural modulus of the laminates prepared with IN2 and IB2 resin is approximately 5.5 GPa and the modulus of Elium is 15% lower compared to the epoxy-based resins. On the other hand, Figure 6c shows that the composite laminates prepared with the IN2 resin present the highest values, approximately 124 MPa, and the composite laminates prepared with both Elium and IB2 present a strength that is 10% lower.

### 3.4. Out-of-Plane Impacts

#### 3.4.1. Perforation Tests

**Figure 7:**
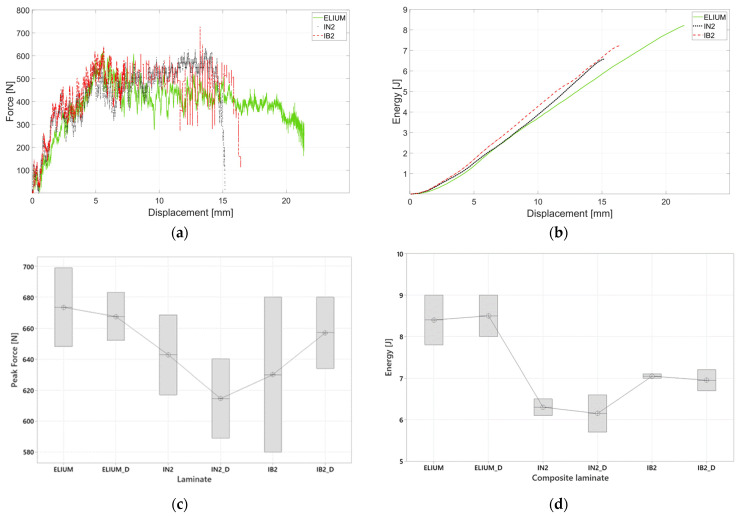
(**a**) Representative force-displacement curves of the drop dart impact tests; (**b**) Representative energy-displacement curves of the drop dart impact tests; (**c**) box plot of the peak force; (**d**) box plot of the absorbed energy.

Perforation tests were carried out on all the composite laminates at an impact energy of 25 J. Figure 7a–d report the summary of the results of the impact testing campaign. Figure 7a displays three representative force-displacement curves. The force-displacement curves present a similar behavior in both initial trends (representative of the stiffness of the plates) and maximum load. This behavior was also shown by Ciardiello et al. [58] when analysing composite laminates made with carbon fibers and resin with different bio contents. They showed that the load-displacement curves have the same initial linear trend and maximum load in drop dart impact tests. This behavior is mainly due to the fiber response that is the same for all the composite laminates up to the fiber failure. In particular, Figure 7a illustrates that the maximum load is reached for all the curves at around 5 mm and then it is maintained for at least 6 mm. This is typical of composite materials made with flax fibers, as shown by Gianmaria et al. [57]. It is noticeable that composite laminate prepared with ELIUM shows a larger displacement. This displacement, observed also in the flexural tests, led to larger absorbed energies as reported in Figure 7b. The boxplot of Figure 7d reports the values of the energy. It can be noticed that the composite prepared with Elium resin presents an absorbed energy that is 50% higher compared to IN2 resin. On the one hand, composite plates prepared with IB2 resin present a value that is 10% higher compared to IN2 resin. Figure 7c shows the boxplot of the peak forces. The ANOVA analysis showed that there is no significant difference among the analysed values; a $P_{value}=0.709$ was found for the peak forces. On the other hand, the end of the ANOVA analysis shows that there is a significant difference in the values of the absorbed energies. A $P_{value}=0.019$ was found for the analysis conducted on the energies. Although the differences in the absorbed energies are quite large, the force-displacement curves do not show significant differences.

#### 3.4.2. Repeated Impacts

**Figure 8:**
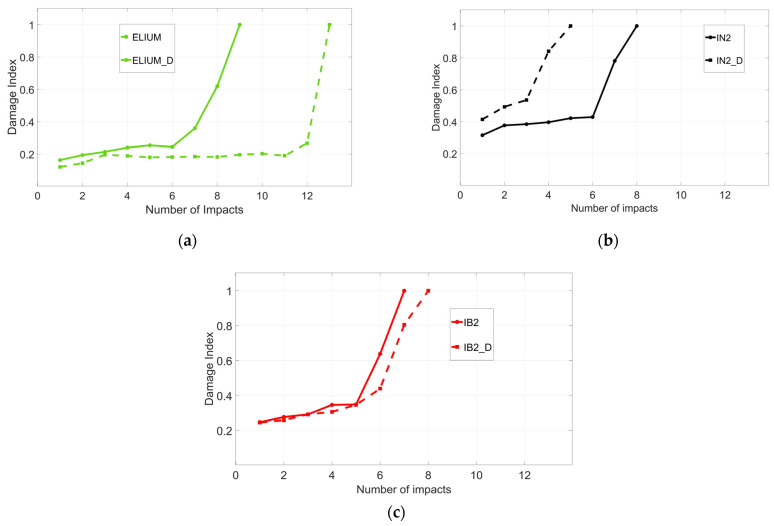
(**a**) DI for Elium laminates; (**b**) DI for IN2 laminates; (**c**) DI for IB2 laminates.

The Damage Index was computed by repeating impact tests on the same laminate and for the six different analyzed laminates at an impact energy of 3.5 J. The aim was to monitor the damage progression during repeated impacts. Figure 8a, Figure 8b and Figure 8c show the computed damage index for the different composite laminates, Elium, IN2, and IB2 resins, respectively. The dashed lines are related to the laminates prepared with dried fabrics while the solid ones refer to the laminates prepared with undried fibers. There is not a tendency that shows a different behavior between dried and undried fabric composite laminates. However, the DI analysis shows again that IN2 resin and IB2 resin present very similar results. For IN2, IB2, and IB2_D specimens, the DI at first impact is 0.3 while for IN2_D it is higher at 0.4. The higher values of DI for specimen IN2_D led to a quicker rupture, after five impacts, while the IN2 specimen fails after eight impacts. The DI for the composite laminates prepared with IB2 resin presents identical damage progression except for the last points, since IB2_D fails after eight impacts while IB2 fails after seven impacts. Composite laminates prepared with Elium resin present a different behavior. The DI after the first impact is below 0.2 for both laminates, thus two times lower than the epoxy-based resin IN2 and IB2. Then, for Elium_D, the DI remains constant up to the 11th impact before failure that occurs after 13 impacts. Elium specimens present a similar behavior up to the 6th impact. Afterwards, the failure occurs at the 9th impact. Overall, the composite laminates prepared with Elium resin present a lower damage progression compared to the epoxy-based resin. This could be due to the larger deformation which ELIUM resin can bear.

**Figure 9:**
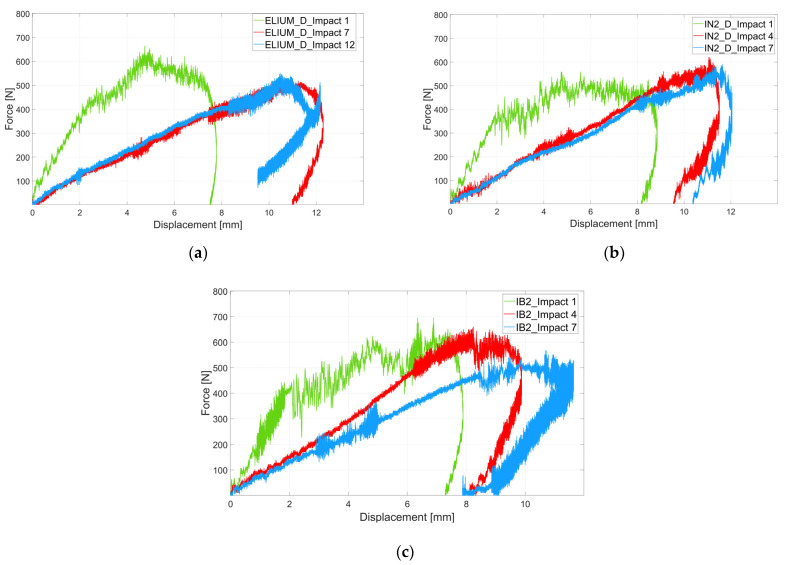
(**a**) Force-displacement curves of repeated test for Elium laminates; (**b**) Force-displacement curves of repeated tests for IN2 laminates; (**c**) Force-displacement curves of repeated tests for IB2 laminates.

Figure 9a–c report three force-displacement during damage progression. In particular, the green curve shows the first impact, the red curve shows an impact between the first and the last impact, and the blue curve the last test before failure. The experimental campaign shows that by increasing the number of impacts, the displacements increase, and the force slightly decreases for ELIUM specimens. On the other hand, the forces remain almost constant for composite laminates prepared with IN2 and IB2 resins. Further, the curves show that a peak force at 4 mm is more recognizable for the ELIUM composite at the first impact. Contrarily, IN2 and IB2 resins, after a first peak of 400 N at 2 mm, present an almost constant trend before the displacement changes the sign, which is a typical behavior of the rebound when the specimen does not reach the perforation.

### 3.5. Failure Surfaces

**Figure 10:**
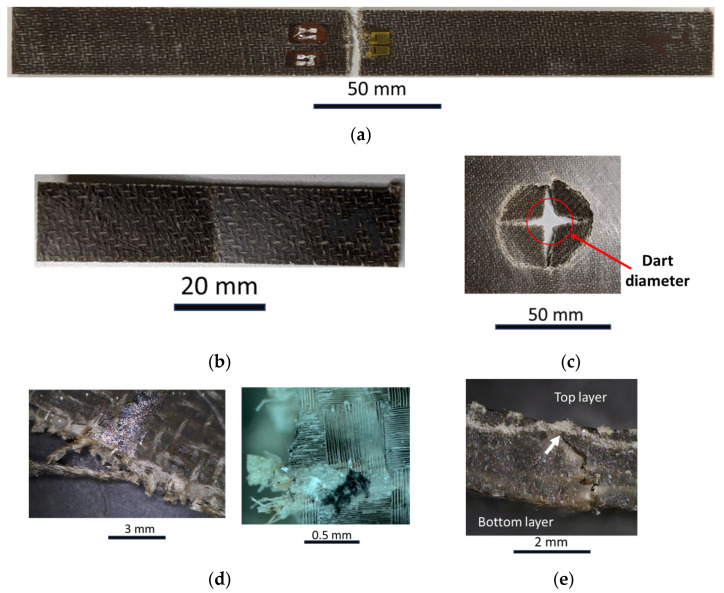
Fracture surfaces of (**a**) tensile specimen; (**b**) flexural specimen; (**c**) impact specimen; (**d**) optical analysis of tensile specimen; (**e**) optical analysis of flexural specimen.

Figure 10a, Figure 10b and Figure 10c depict the representative failure surface of tensile (a), flexural (b), and impact specimens, respectively. Figure 10d,e report the microscopy analysis of the failure surfaces. Figure 10a shows that the tensile failure occurred in the mid-part of the specimen according to the standard recommendations. In this case, the failure occurred close to the strain gauge element, which is still visible in the image. However, all the fractures were close to the midpoint of the specimens and far from the clamping area. The failure surface is quite clear and defined and by considering the microscope analysis of this specimen (Figure 10d); the yarns failed and were pulled out from the matrix. Figure 10d also shows the presence of the resin of the pulled-out fibers that is a sign of good interaction between the matrix and fibers. Figure 10b shows the flexural specimen from the top view, the point where the central pin transmits the load. During this test, the top layer of the specimens undergoes compressive loads while the bottom layers experience tension loads. Figure 10e displays a lateral section of the flexural specimen and how the fracture appears through the thickness. Figure 10e illustrates that the fracture propagates from the bottom layer to the top layer. The top layer was not broken during the test and the tip of the fracture (indicated with the white arrow) stopped when the crack met the top layer, represented by the wavy layer visible in Figure 10e. Finally, Figure 10c illustrates a representative fracture of the impact specimens. Both repeated and perforation impacts presented the same fracture when complete failure was reached. The red circle indicates the external diameter of the spherical dart tip. Although the top layer, close to the tip, undergoes compression load, the fibers present a tension failure due to the dart displacement that tends to separate the layers when final failure occurs, very similar to those obtained in Figure 10d.

### 3.6. Discussion

The mechanical behavior of the three different composites made with three commercial resins shows that composite laminates with reduced environmental impact can be manufactured, thereby reducing the overall carbon footprint of these materials. The summary report of the producer [47,48] of IN2 and IB2 resins illustrates that the bio-based resin IB2 present a carbon footprint that is on average 40% less than IN2 resin system. The acidification impact of IB2 resin is 33% less than IN2. The human toxicity impact of traditional epoxy systems is three times less than the partially bio-based resin. However, the eutrophication impact of bio-based systems is 50% higher than IN2 resin [47,48]. Similarly, although specific information on the life cycle assessment of Elium resin is not available, the environmental impact of this resin is low due to its recyclability properties and its nature as a thermoplastic polymer. The pros and cons reported in Section 3 are summarised in Table 3. The work shows that the drying treatment does not significantly enhance the mechanical properties. For this reason, the drying pre-treatment can be avoided by reducing carbon footprint. The activity illustrates that a moisture content of around 6.1% is found when the environmental relative humidity is between 30% and 40%. Thus, preliminary tests can be carried out to correlate the moisture content starting from the moisture of the environment to avoid, when possible, the heat treatment if the mechanical properties are acceptable for the specific applications. IB2 resin presents a carbon footprint lower than 40% compared to traditional epoxy resin IN2. Thus, IB2 resin can be used in applications where higher tensile and flexural properties are required and to lower the carbon footprint of composites. Also, Elium can significantly lower the carbon footprint of the composite and can be used in applications where higher absorption capabilities are needed.

The work shows that IB2 resin can be used in applications where higher tensile and flexural properties are required. The mechanical properties showed that composite made with IB2 resin present very similar properties compared to IN2 resin. On the other hand, composites made with Elium can be used in applications where larger deformation and higher absorption energies are required. The effects of tensile and flexural properties of epoxy-based composite laminates produced by conditioning the flax fibers at a relative humidity from 0% to 95% were studied by Moudood et al. [40,44]. They reported the change in the tensile strength, tensile modulus, flexural strength, and modulus by conditioning the fibers in a controlled chamber before the infusion. The main outcome of the works is that Young’s modulus decreases for composite materials made with conditioned fibers. Contrarily, the tensile strengths increase for composite laminates fabricated by conditioning the fibers. However, Moudood et al. [40,44] did not report the effective moisture content in the fibers and their analysis was carried out on unidirectional flax fibers. Furthermore, a statistical analysis for the different conditions has not been reported and for composite laminates fabricated with conditioned fibers at a relative humidity of up to 50% the error bars of the strengths and the moduli are overlapping. In contrast, the change in the mechanical properties of composite materials produced with fibers conditioned at a relative humidity of 70% and 95% is significant. The increase of strength for composite materials at a relative humidity of 95% is 10%, while the decrease in Young’s modulus is 26%. The tests carried out in our work showed, through ANOVA, that there are no significant differences in composite laminates fabricated at a relative room humidity between 30% and 40%. Moreover, the results are similar for bio-based and thermoplastic resins. Finally, the dynamic impact properties and the effect of the relative humidity on the properties have never been investigated before.

## 4. Conclusions

The mechanical properties of composite laminates fabricated with dried and undried fibers and three different commercial resins have been studied in this work. The main outcome can be summarized as follows:

1. The flax fabric in the lab environment at a relative humidity between 30% and 40% embeds an average moisture content of 6.1 wt.%.
2. The CT analysis shows that the laminates fabricated with the dried fibers present a porosity close to 0% for all the analyzed cases. IN2 and IB2 resins present a porosity of 0.4% after drying the fibers, whereas they present a porosity of 2.4% and 4.6% by using undried fibers. On the other hand, composite laminates prepared with Elium resin present a value close to 0% for both dried and undried fibers.
3. The tensile tests show that there is no significant difference among Young’s moduli for the different laminates. The average value of the modulus is 8.5 GPa. Composite laminates prepared with Elium present a lower strength compared to IB2 and IN2 laminates, approximately 8% less.
4. The flexural tests showed that composite laminates prepared with Elium resin present a flexural modulus lower than 15% compared to composite laminates prepared with IN2 and IB2-based laminates. While the strength of the laminates prepared with Elium and IB2 is 10% lower compared to the conventional epoxy resin IN2.
5. Impact tests at perforation showed that the laminates prepared with the three different resins and both dried and undried fibers present the same mechanical response. The laminates prepared with Elium resin can absorb higher impact energy due to the larger deformations these laminates can bear. Further, they can better absorb the repeated impacts.

Overall, the experimental activity shows that the presence of moisture, 6.1 wt.%, in the flax fibers led to the presence of voids in the laminates that do not influence the mechanical behavior of the composite laminates. Although the investigated quasi-static and impact properties are representative of the general mechanical behavior of the composite laminates, further tests are needed to assess the mechanical behavior of composites under different environmental conditions. Further studies may involve the fatigue behavior of the composites prepared with dried and undried flax fibers, since composites prepared with undried fibers present higher porosities that can lead to premature failures. Moreover, the environmental conditions could significantly affect the mechanical properties of composites made with different resins.

## Figures and Tables

**Table 1:** Comparison of the mechanical properties of the two investigated resins [47,48,49].

Resin	Elium	IN2	IB2
Density (g/cm^3^)	1.01	1.14	1.12
Viscosity (mPa · s)	100	325	185
Young’s Modulus (GPa)	2.6	3.0	2.8
Tensile strength (Mpa)	56	68	68
Flexural Modulus (Gpa)	2.9	3.3	2.8
Flexural strength (Mpa)	111	120	107

**Table 2:** Total porosities evaluated through computed tomography acquisitions.

Porosities (%)		
Resin	Undried	Dried
Elium	0.0 *	0.0 *
IN2	2.3 (±0.1)	0.4 (±0.05)
IB2	4.6 (±0.1)	0.4 (±0.1)

* Limited by the resolution of the µ-CT.

**Table 3:** Pros and cons of the laminates prepared with IN2, IB2, and Elium resins.

Composite Laminate	Pros	Cons	Possible Applications
IN2 Resin	Highest tensile and flexural properties	Lowest absorbed energy at impact; Lowest resistance to repeated impact; Highest environmental impact; Porosity of 2.4% without drying the fibers.	Applications where higher tensile and flexural properties are required
IB2 Resin	Tensile and flexural properties comparable with traditional epoxy resin; Low environmental impact; Absorbed energies at impact slightly higher than IN2 resin; Same cost of traditional epoxy resin	Porosity of 4.6% without drying the fibers;	Applications where higher tensile and flexural properties are required; Applications were composite with reduced environmental impact is required.
Elium Resin	Good tensile and flexural properties; Low environmental impact; High absorbed energy at impact and good resistance to repeated impacts; Composites free of defects.	Tensile and flexural properties 10% lower compared to the epoxy-based resins. The manufacturing requires resin break to obtain composites free of defects.	Applications were larger deformation and higher absorption energies are required.

## Data Availability

Data will be available on request.
